# Supplementary material for: Above-Filter Digestion Proteomics Reveals Drug Targets and Localizes Ligand Binding Site
Source: J Proteome Res. 2026 Feb 7;25(3):1556–70. doi: 10.1021/acs.jproteome.5c00927 (PMC12973292; doi:10.1021/acs.jproteome.5c00927)
Supplement: Supplementary file 1 [file pr5c00927_si_002.pdf]

Above-Filter Digestion Proteomics reveals drug targets and localizes ligand binding site

Supplementary material

Supplementary figure 1

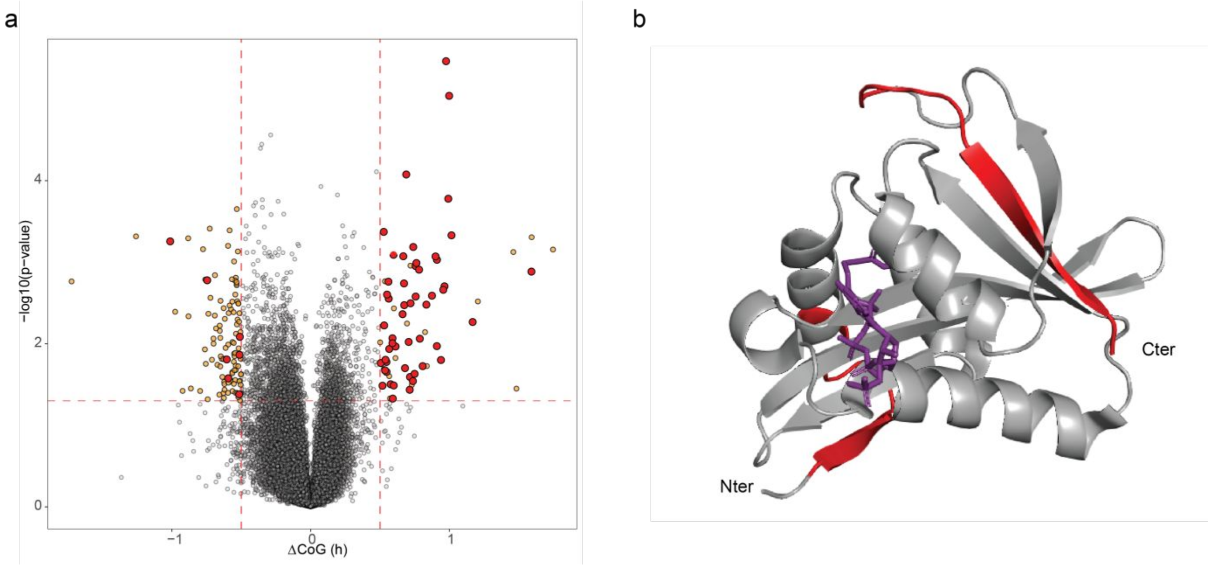

Supplementary figure 1. (a) Volcano plot for AFDIP showing all peptides having a significant center of gravity shift in orange (cut-off values are the same as in Figures 2-5) and peptides belonging to proteins involved in CoA metabolism and production in red. (b) NAA50 complex with AcCoA. Shifting peptides used for CoM calculations colored red, AcCoA molecule in purple.

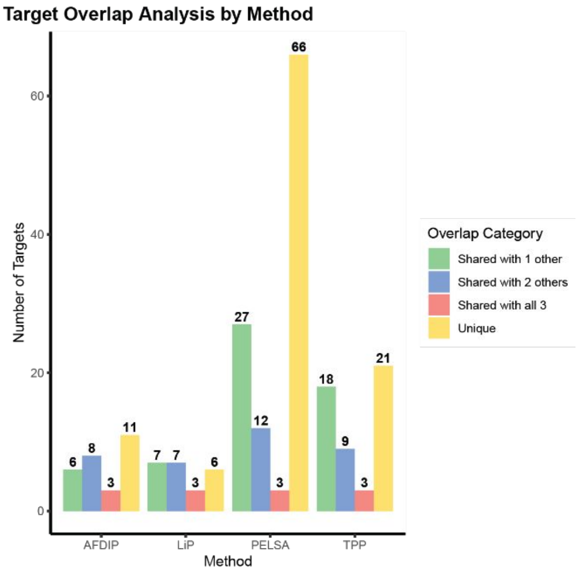

Supplementary figure 2. Distribution of targets identified by each method (AFDIP, LiP, TPP, PELSA) categorized by overlap patterns: unique (yellow), shared with 1 other method (green), shared with 2 other methods (blue), or shared with all 3 other methods (red). Numbers indicate target counts per category.
